# Clinico-Epidemiological Profile of Intussusception Among Children Younger than 2 Years in Karnataka, India: A Multicenter Hospital-based Surveillance Study

**DOI:** 10.1007/s12098-025-05719-z

**Published:** 2025-09-11

**Authors:** Divya Arvind Prabhu, Hassan Sreenivasamurthy Rajani, Mahantashetti Niranjana, Jayatheertha Joshi, Sudhamshu Kalasapura Chandrashekar, Attibele Mahadevaiah Shubha, Santosh Kurbet, Kamalakshi G. Bhat, Jakati Vishal, Mohammad Rabbani, Harika A. Marri, Vijay Kumar, Veena Ganesh Kamath, Rajkiran Srinivas Raju, Varsha Sudhir Chaudhary, Anupama Machathi, Namrata Kharat, Poovarasan Kannan

**Affiliations:** 1 Department of Community Medicine, Kasturba Medical College, Manipal, Manipal Academy of Higher Education, Manipal, Karnataka, India; 2 Department of Pediatrics, JSS Medical College, JSS Academy of Higher Education and Research, Mysore, Karnataka, India; 3 Department of Pediatrics, JN Medical College, Nehru Nagar, Belagavi, Karnataka, India; 4 Department of Pediatric Surgery, Father Muller Medical College, Mangalore, Karnataka, India; 5 Department of Pediatric Surgery, JSS Academy of Higher Education and Research, Mysore, Karnataka, India; 6 Department of Pediatric Surgery, St. John’s National Academy of Health Sciences, Bengaluru, Karnataka, India; 7 Department of Pediatric Surgery, JN Medical College, Nehru Nagar, Belagavi, Karnataka, India; 8 Department of Pediatrics, Kasturba Medical College, Mangalore, Manipal Academy of Higher Education, Manipal, Karnataka, India; 9 Department of Pediatric Surgery, Kasturba Medical College, Manipal, Manipal Academy of Higher Education, Manipal, Karnataka, India; 10 Department of Community Medicine, Christian Medical College, Vellore, Tamil Nadu, India

**Keywords:** Intussusception, ROTASIIL, Rotavirus, Surveillance, Immunization

## Abstract

**Objectives:**

Immunization with conventional rotavirus vaccines (RVVs) has been reported to intensify the risk of intussusception (IS). However, the risk of IS following vaccination with the recently introduced ROTASIIL is not clearly understood. The aim of this study was to describe the clinical and epidemiological profile of IS among children after vaccination with RVV.

**Methods:**

A hospital-based multicenter observational study was carried out at five private medical college-based tertiary care hospitals in Karnataka, India, over a 30-mo period. Children younger than 2 y admitted with confirmed IS were enrolled. Data were collected on demographics, clinical features, and vaccination history.

**Results:**

Of 196 children with confirmed IS, 153 (78%) had received ROTASIIL. The median age (interquartile range [IQR]) of the participants was 9 (6–14) mo, and 69% were male. The median age (IQR) at receiving first, second, and third dose of RVV was 7 (6–9) wk, 12 (11–14) wk, and 17 (16–20) wk, respectively. Of the participants, 91.8% had ileo-colic IS and the IS was resolved by imaging-guided reduction in 78.6%. Two, three, and eleven cases of IS were reported within 21 d after receiving the first, second and third dose of RVV, respectively.

**Conclusions:**

This study provides baseline epidemiological data on IS among children younger than 2 y. Although, no definite association between ROTASIIL and IS was noted, this study provides health authorities with information on the risks and benefits of ROTASIIL in a real-world setting.

## Introduction

Intussusception (IS) in children is a common surgical emergency with an incidence of 26–38 cases per 100,000 live-births [1]. In the absence of a robust registry on surveillance of IS, the exact incidence remains unclear. Surveillance studies have found wide geographic variation in the incidence of IS. The incidence has been reported to be as high as 254 cases per 100,000 births in some South Indian states, whereas the reported incidence is considerably lower (17.7 cases per 100,000 births) in North Indian states [2]. The South Indian states of Kerala and Tamil Nadu have recorded highest geospatial clustering of IS [3].

ROTASIIL was introduced in Karnataka and integrated into the Universal Immunization Program (UIP) on August 26, 2019 [4]. Monitoring adverse events following immunization through clinico-epidemiological studies is recommended to ensure recipient safety [5]. This study was conducted after the introduction of ROTASIIL in Karnataka under the UIP.

## Material and Methods

**Fig. 1:**
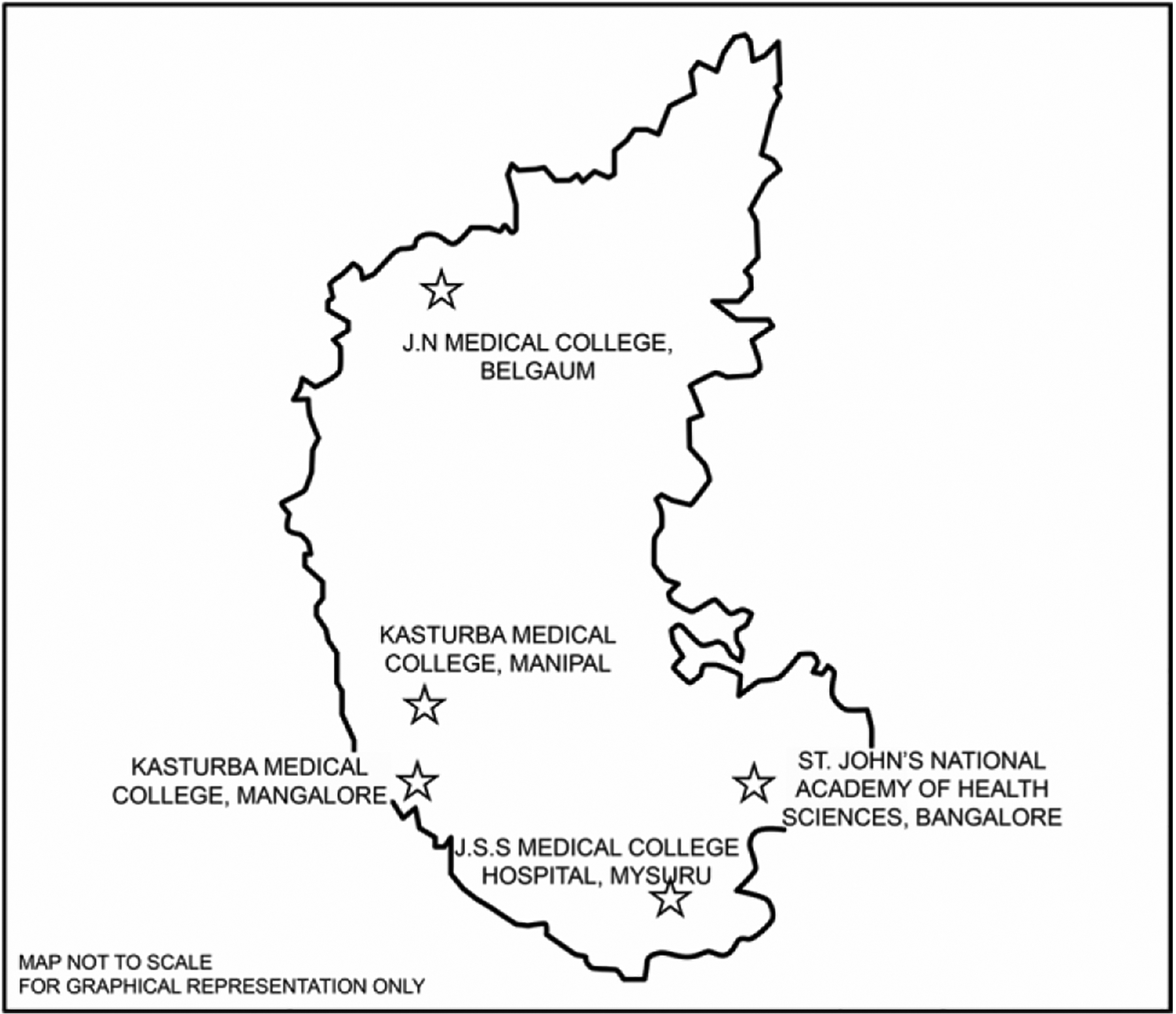
Location of the five intussusception sentinel surveillance sites in Karnataka

The overall study design and methods are outlined in the introductory article in this supplement [6]. The study included children under 2 y of age, admitted to the pediatric or pediatric surgery wards in five private medical college-based tertiary care hospitals in Karnataka over a 30-mo period between June 2020 and December 2022. The locations of the five study sites are shown in Fig. 1. Potential participants were identified by surveying inpatient ward, surgical theater, and radiology logs in coordination with the pediatricians, pediatric surgeons, radiologists, and the medical records department of each institution. Eligible children were enrolled after obtaining informed consent from the parent or guardian, as described in the introductory article [6]. The onset of IS was defined as the date of the first clinical manifestation, as reported by the parent or caregiver.

The study was approved by the Institutional Review Board of the coordinating institution and the research ethics committee of each participating institution.

Demographic and clinical data were collected using a structured case report form, as described in the introductory article in this supplement [6]. The data were analyzed using Stata version 16.1 [7] and SPSS version 15 [8], as described in the introductory article [6].

## Results

**Fig. 2:**
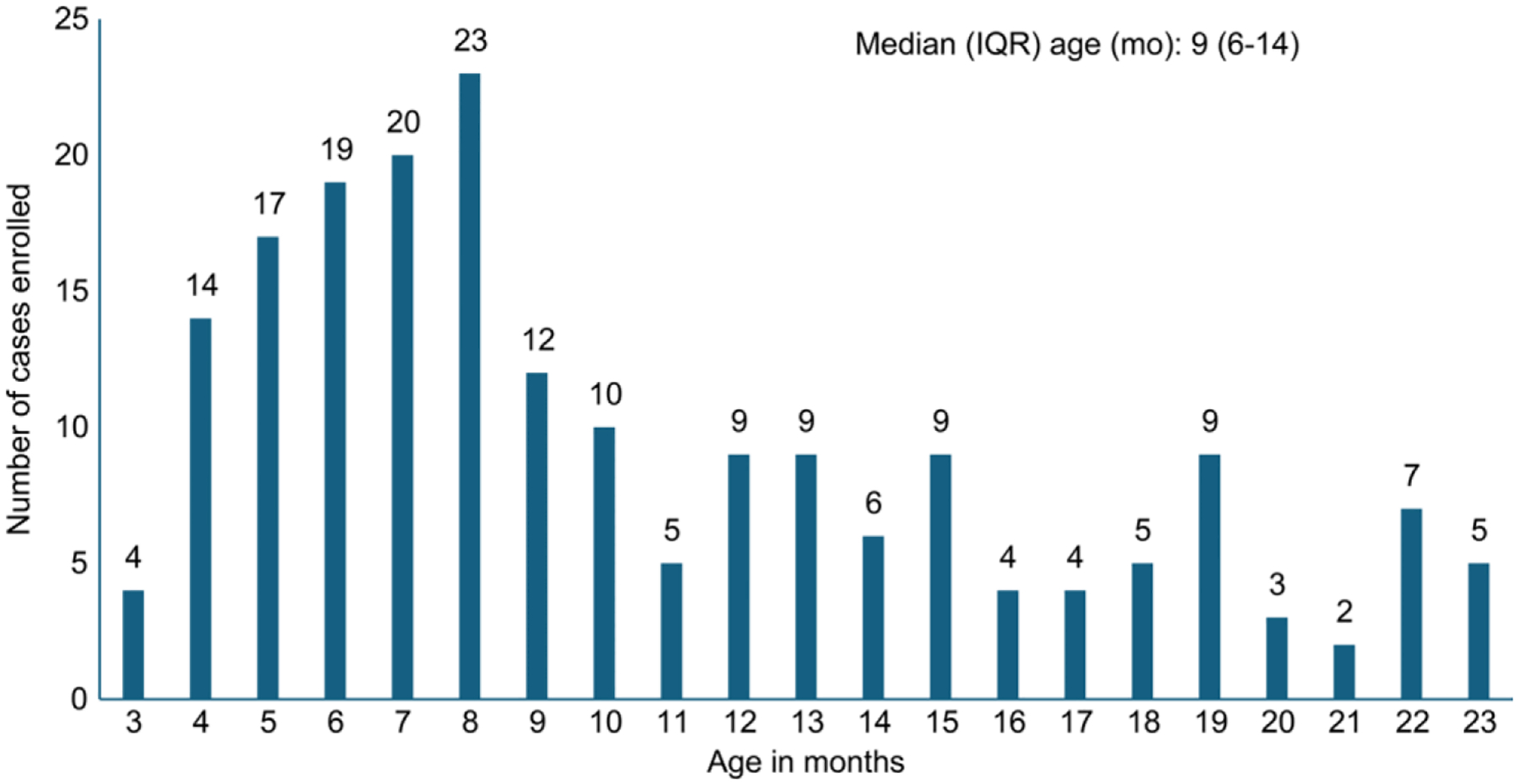
Age distribution of children with intussusception

Over a period of 30 mo, 326 children with IS were screened and 196 (60%) who satisfied the eligibility criteria were enrolled at the five study centers. Children presented at a median age of 9 mo (IQR: 3–14 mo), with 4 (2%) in 0–3-mo age group and 79 (40.3%) in the 7–12-mo age group, with a male predominance (69%). The age distribution of children presenting with IS is shown in Fig. 2. Most of the children (78%) had received at least one dose of rotavirus vaccine RVV, with 85.6% of all vaccinated children having received all the three doses. The first, second, and third doses of RVV were administered at median age of 7 wk (Interquartile range; IQR: 6–9 wk), 12 wk (IQR: 11–14 wk), and 17 wk (IQR: 16–20 wk), respectively.

Of the participants, 67 (41.1%) were weaned off breast-feeds before the age of 6 mo. Approximately half (49%) of the mothers of children had an educational level of higher secondary school or above, and 61% were aged 26–35 y, with a mean age of 33.3 ± 20.6 y. Most mothers (92%) were of lower or middle socioeconomic status, based on the wealth index (Table 1). The incidence of IS showed seasonal variation, peaking in the winter months, from January to March (Table 1).

Abdominal pain (82.7%) was the most common presentation, followed by vomiting (81.3%), blood in the stool (48.7%), and diarrhea (22.3%). The median interval between the onset and presentation to the hospital was 1 d (IQR: 0–2 d), with three-quarters of the children (*n* = 148, 75.5%) being brought to the hospital 1–3 d after the onset. Ileo-colic IS was the commonest location (*n* = 180, 91.8%), and the median duration of hospitalization was 2 d (IQR: 2–3 d). More than three-quarters (*n* = 154, 78.6%) of the children were treated by pneumatic or hydrostatic reduction; 36 (18.4%) underwent surgery following failed pneumatic or hydrostatic reduction, and 6 underwent surgery ab initio, without attempting pneumatic or hydrostatic reduction. Three of the five study centers used fluoroscopy-guided pneumatic reduction, and the other two centers used hydrostatic saline reduction. In the surgical group, the most common complication was wound infection. No significant difference in the clinical manifestations was noted between the vaccinated and unvaccinated children. None of the participating centers reported any deaths (Table 2).

The treatment modality did not differ significantly according to the mode of presentation of IS or vaccination status (Table 2). Of the children presenting with IS, 154 (78.6%) were treated successfully with pneumatic or hydrostatic reduction, whereas 6 (3.1%) children were directly taken up for surgical methods of reduction. Two cases of IS were diagnosed within 2 d of administration of the first dose of RVV, and four cases of IS were reported more than 21 d after the first dose but before receiving the second dose. Two, one, and 15 cases were reported 1–7 d, 8–21 d, and more than 21 d, respectively, after receiving the second dose of RVV but prior to receiving the third dose. Seven and four cases were reported 1–7 d and 8–21 d, respectively, after receiving the third dose of RVV, whereas all the remaining cases occurred beyond the at-risk period of 21 d following the last dose of RVV (Table 1).

Almost all children, 99% (*n* = 194), were discharged after a median duration of hospitalization of 2 d (IQR: 2–3 d). One child was transferred to another health care facility at the request of the family, and another child was discharged against medical advice. No deaths occurred during hospitalization.

## Discussion

This multicenter cross-sectional surveillance study carried out at the five private tertiary care hospitals across Karnataka enrolled 196 children younger than 24 mo with IS. The finding of a male preponderance (69%) in this study is consistent with those of other studies of children with IS conducted in the Indian subcontinent (57–66%); however, the reason for this male preponderance remains unexplained [2–4, 9]. In this study, the children presented at a median age of 9 mo, with 40.3% aged 7–12 mo, with a slightly higher age distribution than those reported in previous studies. Geospatial clustering of IS in Southern India has been reported previously and may be partially explained by the higher literacy rates, prompt referral, good access to healthcare, and higher number of pediatricians and pediatric surgeons in the region compared with Northern India [10]. Myat et al. [11] reported that 41.9% of children with IS were aged 5–7 mo, whereas Jena et al. [12] reported that 57.1% were aged 4–8 mo. Surveillance studies have reported clustering of cases of IS in the United States and Australia within 21 d after vaccination with RVV [13–15]. However, no such clustering was observed in this study. In this study, no children aged 0–2 mo were admitted with IS, and only four children were aged less than 3 mo. Multiple previous studies conducted in South Asian countries (including Bangladesh, India, China, Nepal, Pakistan, and Vietnam), Fiji; and Latin America have also reported a low incidence of IS in infants aged 0–3 mo [16–20]. These findings strongly support the World Health Organization’s recommendation of administering the initial two doses of RVV at 6 and 10 wk of age, as the IS incidence is extremely low in this age group. IS has been hypothesized to be linked to intestinal replication of rotavirus. Several studies conducted in India and other low- and middle-income countries have shown lower vaccine efficacy than that in high-income countries, suggesting decreased intestinal viral replication and a lower risk of IS [21]. The simultaneous administration of RVV and oral polio vaccine does not affect the risk of developing IS [21]. The median age at administration of the first, second, and third doses of RVV in this study are similar to those reported to the episode in another nationwide study conducted in India by Reddy et al. [22].

Seasonal clustering of IS was observed in present study during January to March, which differs from the seasonal clustering reported in other Indian studies conducted by Das et al. [10] (March to June) and Jena et al. [12] (April to July). In contrast, Pradhan et al. [23] reported bimodal peaks in the number of cases in November to January and March to June. Notably, several other studies did not show seasonal clustering of IS [2, 12, 22, 23], although crowding in cooler climates could favor oral transmission of rotavirus. Pradhan et al. [23] reported no significant difference in presenting symptomatology between vaccinated and unvaccinated children, akin to present study. The median time interval between the onset of symptoms and presentation to the hospital was 1 d, which is at par with the studies by Das et al. [10], Liu et al. [16], and Pradhan et al. [23]. Most cases of IS were ileo-colic, consistent with the findings of several other Indian studies [2, 12, 22, 23]. The predominant involvement of the ileo-colic region is presumed to be triggered by idiopathic Peyer’s patch hypertrophy in the terminal ileum [24]. The median duration of hospitalization in this study was 2 d, which is consistent with that reported in a study by Jena et al. [12] but shorter than that reported by Liu et al. [16] (4 d) and Das et al. [10] (3 d). The median duration of hospitalization is generally shorter in those undergoing non-invasive imaging-guided reduction (2 d) than in those undergoing surgery (6 d) [2].

No deaths were reported in the study cohort. Studies conducted by Das et al. [10], Liu et al. [16], and Pradhan et al. [23], have reported case fatality rates ranging from 0.5 to 1%. Higher mortality rates have been reported in children with IS who present late with intestinal devitalization and/ or fulminant sepsis [16, 23].

The role of early weaning and introduction of complementary feeds in the pathogenesis of IS has also been investigated. In a case-control study conducted in Italy, Pisacane et al. [25] reported higher IS rates in exclusively breastfed infants than in infants who were not breastfed or were weaned early. Similarly, in a study conducted in Nigeria, Kesiena et al. [26] reported higher IS rates in exclusively breastfed infants than in those weaned to a maize-based weaning porridge. Furthermore, Johnson et al. [27] reported higher rates of IS in infants weaned to cow’s milk than in those weaned to soya-based formula. These dietary factors need to be evaluated further to evaluate the potential role of food allergy in the causation of IS.

Strengths of this study include its multicenter prospective design for surveillance across a wide geographic area, adherence to standard case definitions, rigorous screening of electronic data, and adherence to a uniform protocol across all study sites. The coordinating center conducted stringent monitoring to ensure data completeness and accuracy, thus minimizing the occurrence of classification and data abstraction errors.

This study also has some limitations. All five study sites were tertiary, private medical college-based hospitals that drew patients from a wide catchment area. This may limit the generalizability of the findings, and as the study sites did not have a clearly defined catchment population or referral pattern, it is not possible to estimate the incidence of IS in the population of children aged under 2 y in Karnataka from the study data.

## Conclusions

This study provides valuable baseline epidemiological data on IS among children aged under 2 y following the introduction of ROTASIIL under the UIP in Karnataka. The findings are reassuring because the study did not find evidence of an association between vaccination with RVV and IS. To facilitate the post-marketing surveillance and formulation of public health policy on rotavirus vaccination, further large multicenter surveillance studies covering a wider geographic area are warranted.

## Supplementary Material

RIS Karnataka Anonymized Dataset

RIS_Karnataka_Data_dictionary

## Figures and Tables

**Table 1:** Baseline and clinical characteristics of children younger than 2 y admitted to the five study centers in Karnataka with intussusception (*N* = 196)

Characteristic	Value
Sex, n (%)	
Male	135 (69.0)
Female	61 (31.0)
Rotavirus vaccination status, n (%)	
Unvaccinated	43 (21.9)
1 dose	6 (3.1)
2 doses	16 (8.2)
3 doses	131 (66.8)
Interval between last dose of rotavirus vaccine & onset of intussusception, n = 194 (%)^a^	
Unvaccinated	43 (21.9)
1–7 d	11 (5.6)
8–21 d	5 (2.6)
> 21 d	135 (68.9)
Duration of exclusive breastfeeding, n (%) (*N* = 163)	
< 6 mo	67 (41.1)
≥ 6 mo	96 (58.9)
Wealth index, n (%)	
Lower	100 (51.0)
Middle	80 (40.8)
Upper	16 (8.2)
Maternal age (y), n (%) (*N* = 179)	
≤ 25	64 (35.6)
26–35	109 (61.0)
≥ 36	6 (3.4)
Incidence of intussusception by season (mean ± SD)	
January to March (Winter)	18.33 ± 0.57
April to June (Summer)	17.00 ± 2.64
July to September (Monsoon)	9.00 ± 3.36
October to December (Autumn)	13.5 ± 3.69
Maternal education, n (%) (*N* = 179)	
No formal education	2 (1.0)
Primary	33 (16.8)
Secondary	48 (24.5)
Higher secondary	54 (27.6)
Graduate and above	42 (21.4)
Unknown	17 (8.7)
Clinical presentation, n (%)	
Abdominal pain (*N* = 191)	158 (82.7)
Vomiting (*N* = 192)	156 (81.3)
Blood in stool (*N* = 193)	94 (48.7)
Diarrhea (*N* = 193)	43 (22.3)
Fever (*N* = 190)	24 (12.6)
Time from onset to hospital admission (d), median (IQR)	1 (0–2)
Time from onset to hospital admission, n (%)	
< 1 d	33 (16.8)
1–3 d	148 (75.5)
> 3 d	15 (7.7)
Location of intussusception, n (%)	
Ileo-colic	180 (91.8)
Ileo-ileal	7 (3.6)
Colo-colic	3 (1.6)
Unknown	2 (1.0)
Multiple sites	4 (2.0)
Treatment method, n (%)	
Pneumatic or hydrostatic imaging-guided reduction	154 (78.6)
Failed UGR followed by laparotomy and manual reduction	36 (18.4)
Direct laparotomy, manual reduction without intestinal resection	4 (2.0)
Laparotomy with intestinal resection	2 (1.0)

*IQR* Interquartile range; *SD* Standard deviation; *UGR* Ultrasound-guided reduction

a One child experienced intussusception on the day of vaccination, while another was vaccinated subsequent

**Table 2:** Presentation and clinical course of children younger than 2 y admitted to the five study centers in Karnataka with intussusception according to rotavirus vaccination status (*N* = 196)

Variable and Category	Vaccinated *n* (%) (*N* = 153)	Unvaccinated *n* (%) (*N* = 43)	*p*
***Clinical manifestation***			
Vomiting^a^ (*N* = 192)			0.820^b^
Yes	123 (80.9)	33 (82.5)	
No	29 (19.1)	7 (17.5)	
Abdominal pain^a^ (*N* = 191)			0.240^c^
Yes	123 (80.9)	35 (89.7)	
No	29 (19.1)	4 (10.3)	
Blood in stools^a^ (*N* = 193)			0.216^b^
Yes	78 (51)	16 (40)	
No	75 (49)	24 (60)	
Diarrhea^a^ (*N* = 193)			0.415^b^
Yes	36 (23.5)	7 (17.5)	
No	117 (76.5)	33 (82.5)	
Fever^a^ (*N* = 190)			0.420^c^
Yes	21 (13.9)	3 (7.7)	
No	130 (86.1)	36 (92.3)	
Constipation^a^ (*N* = 192)			0.767^b^
Yes	6 (4)	2 (5)	
No	146 (96)	38 (95)	
***Treatment modality***			0.065^c^
Imaging-guided hydrostatic/pneumatic reduction	119 (77.8)	35 (81.4)	
Surgery with manual reduction	1 (0.6)	3 (7)	
Intestinal resection	2 (1.3)	0	
Mixed	31 (20.3)	5 (11.6)	

a Excluding those with missing information,

b Calculated using the chi-square test,

c Calculated using Fisher’s exact test
